# Investigation of parameters governing damage resistance of nematic liquid crystals for high-power or peak-intensity laser applications

**DOI:** 10.1038/s41598-019-52305-3

**Published:** 2019-11-11

**Authors:** T. Z. Kosc, A. A. Kozlov, S. Papernov, K. R. P. Kafka, K. L. Marshall, S. G. Demos

**Affiliations:** Laboratory for Laser Energetics, University of Rochester, 250 East River Road, Rochester, NY 14623−1299 USA

**Keywords:** Liquid crystals, Nonlinear optics

## Abstract

We investigate the damage resistance of saturated and unsaturated liquid crystals (LC’s) under a wide range of laser excitation conditions, including 1053-nm pulse durations between 600 fs and 1.5 ns and nanosecond pulse excitation at 351 nm and 532 nm. This study explores the relationship between the LC’s resistance to laser-induced breakdown (damage) and the electronic structure (*π*-electron delocalization) of the constituent molecules. The laser-induced damage threshold at all wavelengths and pulse durations was consistently higher in saturated materials than in their unsaturated counterparts. The wavelength’s dependence in the results suggests that the energy coupling process that leads to laser-induced breakdown is governed by the energy separation between the ground state and the first and second excited states, while the pulse duration’s dependence in the results reveals the important role of electron relaxation between the excited states. A qualitative description was developed to interpret the experimental observations.

## Introduction

Liquid crystals (LC’s) have anisotropic optical properties that make them ideal materials from which to construct either passive or active devices that offer polarization, phase, or intensity control. Even though LC’s are commonly associated with the display industry^1^, other common applications include retarders^2^, spatial light modulators (SLM’s)^3^, active lenses^4^, and privacy windows^5^. SLM’s are particularly important devices because of their ability to function as an optical light valve that can be employed in numerous ways, including applications in currently established or emerging applications in the THz regime^6^ and development of next-generation metal additive manufacturing via large-aperture laser melting^7^.

A less widely known application for LC devices is for spectral and polarization control in large-aperture inertial confinement laser systems. LC circular polarizers and wave plates have been key components in the near-infrared portion of the 351-nm, 1-ns, 40-TW OMEGA laser at the Laboratory for Laser Energetics (LLE) for over 30 years^8^. As with most optical materials incorporated in such laser systems, the laser-induced damage threshold (LIDT) of the material (or optical component) is a key factor in determining its suitability and performance parameters. To date, LC device performance of the OMEGA laser has been largely free of adverse performance issues^9^, but efforts are underway to extend the viability of LC optics to higher fluences (>10 J/cm^2^ at 1053-nm, 1-ns), shorter pulse lengths (<100 ps), and shorter wavelengths (351 nm). Success in this parameter space could be translated to higher-average-power or higher-peak-power laser systems to expand science frontiers and enable new laser-based applications^10^.

Laser-induced damage is commonly determined by the formation of an observable material modification, which is caused by the deposition of laser energy into the material. The energy-coupling mechanisms are expected to be largely dependent on the electronic structure of the material, the presence of absorbing defects structures, and the associated excitation (laser) parameters. Laser-induced damage threshold values of commercially available LC compounds and mixtures were first measured by Jacobs^8^ in 1988 (using nanosecond laser pulses at 1053 nm), and a second limited study compared the behavior of an unsaturated LC compound, 5CB, and its saturated analog, ZLI-S-1185 (4-octylcyanobicyclohexyl)^11^. Different regimes of laser-induced damage at IR wavelengths—continuous wave and pulsed irradiation at 100 Hz—were explored for applications in space photonics^12^ and Thomson diagnostics^13,14^.

This work aims to provide baseline measurements on the LIDT’s of currently available LC’s as a function of their chemical structure and extend the limited available knowledge on LC damage thresholds for nanosecond pulses at 1053 nm to both the subnanosecond and nanosecond pulse length regimes at 527 nm and 351 nm. Several nematic LC materials were selected to explore the effect of varying degrees of *π*-electron delocalization and electron density on their damage thresholds. Delocalized *π* electrons are found in unsaturated (e.g., benzene-like) carbon rings with double bonds, and their presence shifts the electronic absorption edge toward longer wavelengths. Saturated compounds have carbon rings with only single bonds, which essentially eliminate electron delocalization and cause the absorption edge to be shifted toward shorter wavelengths. The wide-ranging series of LC materials studied in this work are shown in Table 1. LC materials with the highest degree of *π*-electron delocalization include the well-known cyano-biphenyl (two unsaturated hydrocarbon rings) LC materials such as 5CB (4-pentyl-cyanobiphenyl or K-15), the eutectic mixture E7, and the 60/40 mixture of two phenyl benzoate ester compounds (PPMeOB and PPPOB) used on the OMEGA laser. The phenylcyclohexane-based mixture ZLI-1646 (Merck) is composed of both unsaturated (benzene) and saturated (cyclohexane) rings. Other evaluated materials included a perfluorinated alkyl LC mixture (Merck MLC-2037) and an isothiocyanate LC compound synthesized by R. Dabrowski (Military University of Technology, Warsaw, Poland). The results provide insight on the damage-initiation mechanisms in LC’s, guidance for the possible implementation of future applications in high-power and/or peak-intensity systems, and direction on required optical and chemical properties for the development of highly damage resistant *solid-state* LC materials (e.g., polymer LC’s and glassy LC’s)^15,16^.

**Table 1:** Nematic LC’s used in this study. Materials are designated as saturated and unsaturated with the symbol S or , respectively, in the molecular structures in the first column. Note that the compounds in ZLI-146 contain both saturated and unsaturated ring structures. Transmission measurements were performed on a Perkin-Elmer Lambda-900 spectrophotometer, while the LC materials were in the isotropic state^26^. The absorption edge is defined at *T* = 98%.

	Name	Supplier	Absorption Edge	Material
	1550C	Dabrowski^∧^	294 nm	Pure
	MLC-2037	Merck	306 nm	Mixture
	ZLI-1646	Merck	324 nm	Mixture
	PPMeOB/PPPOB	LLE*	345 nm	Mixture
	5CB	EM Chemical	377 nm	Pure
	E7	EM Chemical	385 nm	Mixture

^∧^Isothiocyanate compound synthesized by R. Dabrowski, Military University of Technology, Warsaw, Poland.

*A 60/40 mixture of two phenyl benzoate ester compounds used on the OMEGA laser and synthesized at LLE.

## Results

Laser-induced breakdown (damage) thresholds were investigated as a function of pulse length, wavelength, and chemical composition. The effect and influence of a material’s chemical composition on its laser-induced damage threshold can in part be correlated to its UV absorption edge, which shifts to the red with increased *π*-electron delocalization. Therefore, in this work, the pulse length and wavelength dependence of the LIDT are studied as a function of the UV absorption edge of each material.

### Pulse-length dependence at 1053 nm

**Figure 1:**
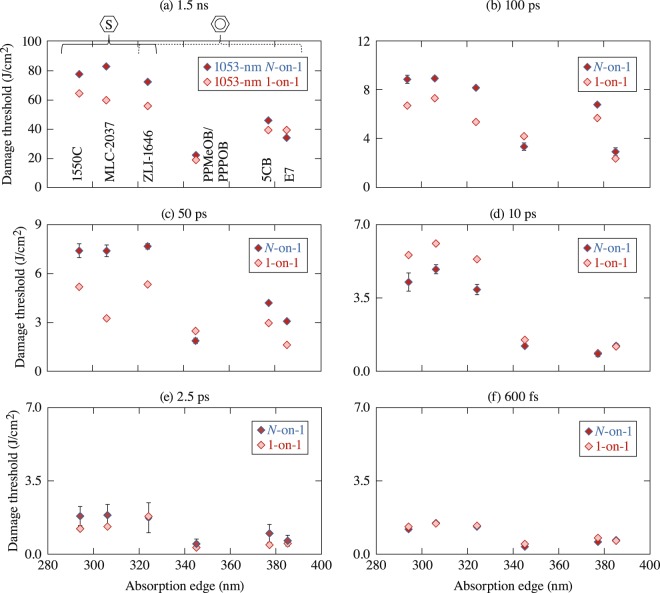
The 1-on 1 and *N*-on-1 LIDT values are plotted as a function of the UV absorption edge at various pulse lengths. The compound names and brackets identifying the saturated, unsaturated, and mixed materials apply to all figures. Materials with completely saturated or mostly saturated carbon rings have absorption edges <330 nm, with correspondingly higher damage thresholds. Errors are of the order of the data marker unless error bars are shown.

The dependence of the LIDT at 1053 nm as a function of the laser pulse duration was investigated at six different pulse lengths (*τ*): 600 fs, 2.5 ps, 10 ps, 50 ps, 100 ps, and 1.5 ns. The 1-on-1 and *N*-on-1 LIDT values are plotted in Fig. 1 as a function of each material’s UV-absorption edge. The saturated materials have an absorption edge <330 nm, and LIDT data for saturated and unsaturated materials can be differentiated easily. The LIDT values determined in this work for three common commercial LC materials (E7, 5CB, and ZLI-1646) were higher than those determined for the same materials in 1988^8^, which can be attributed to advances in chemical purification processes applied to commercial LC materials in general. Of notable significance are the data at 1.5 ns, where the LIDT values of saturated LC’s approach those of bare fused silica^17^.

Figure 1 presents the measured LIDT values as a function of the UV absorption edge of each material. The compounds are identified in Fig. 1(a), where brackets separate the saturated and unsaturated materials. The brackets overlap above the one mixed material. The results suggest that, in general, saturated materials exhibit a 2× to 4× higher LIDT than unsaturated compounds independent of pulse length. The results also show that, at pulse lengths ≥50 ps, the *N*-on-1 LIDT exceeds the 1-on-1 LIDT for most materials. This increase in LIDT with pre-exposure to laser pulses, commonly referred to as laser “conditioning,” indicates the presence of a laser-induced material modification that leads to improved material performance (this effect will be considered in more detail in the Discussion). In contrast, for both saturated materials and the unsaturated mixture PPMeOB/PPPOB at 10 ps, the *N*-on-1 LIDT is *lower* than the 1-on-1 LIDT, an effect commonly referred to as “incubation.” Neither conditioning nor incubation is strongly observed at either 600 fs or 2.5 ps. The same vertical scale was used for Fig. 1(d–f) to help recognize the similar LIDT values for unsaturated materials.

**Figure 2:**
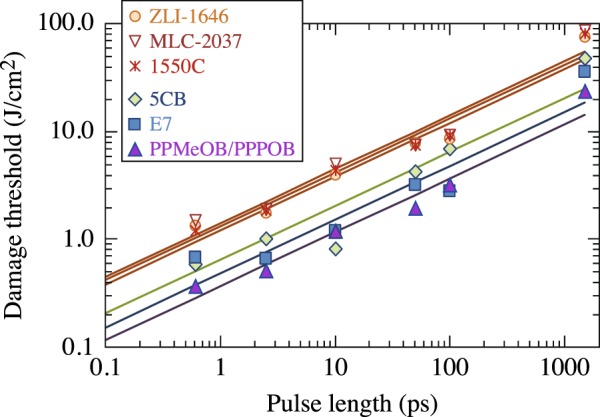
*N*-on-1 LIDT values for saturated and unsaturated LC materials are plotted as a function of pulse length. A fit based on *τ*^0.5^ is shown for each material. The *R*^2^ values range between 0.90 (E7) and 0.96 (PPMeOB/PPPOB, 1550C, and ZLI-1646).

A clear pulse-length dependence emerges from the *N*-on-1 LIDT results plotted in Fig. 2 on a log–log scale and fit as a function of pulse length using *τ*^*x*^ power dependence, where *x* = 0.5. Stuart showed that for dielectric materials, the *τ*^0.5^ power scaling (although experimentally observed to vary between 0.3 < *x < *0.6) is valid for pulse lengths greater than 20 ps, where thermal diffusion effects govern the damage-initiation process^17^. Defects or defect states, in particular, absorb laser irradiation which, in turn, leads to free electrons and ionization of material^18–20^. As pulse lengths decrease below 10 ps, multiphoton ionization starts to contribute to electron production, and in the subpicosecond range, multiphoton ionization becomes the dominant damage initiation process (although avalanche ionization can significantly contribute at the later state of energy deposition). The approximate *τ*^0.5^ dependence has also been observed in biological materials by Loesel^21^ and Oraevsky^22^. At this time, we consider the fact that LC LIDT’s at pulse lengths <50 ps still follow the *τ*^0.5^ trend reasonably well as coincidental. The fit for saturated materials is stronger (*R*^2^~0.96), and the three samples with the lowest absorption edges behave very similarly.

**Figure 3:**
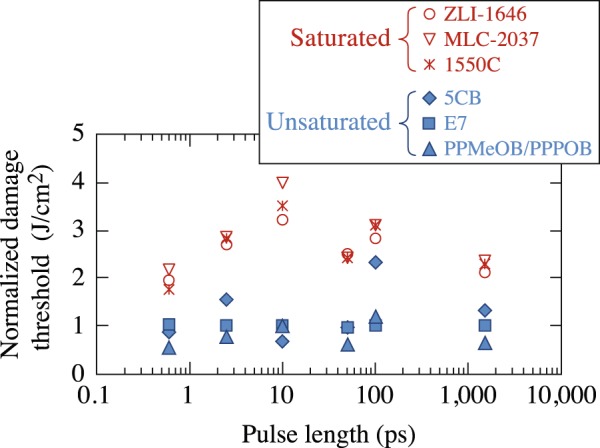
The relative difference between *N*-on-1 LIDT’s of saturated and unsaturated compounds is dependent on pulse length. Data are normalized to results obtained for the unsaturated cyanobiphenyl LC mixture E7. At 10 ps, there is a substantial increase in the difference between the LIDT of the two materials, which may suggest a change in the mechanism for laser-induced damage.

To better quantify the relative difference in LIDT between saturated and unsaturated LC materials, their value at each laser pulse length was normalized to the LIDT’s of the cyanobiphenyl LC mixture E7, a common unsaturated LC mixture formulation. Results shown in Fig. 3 indicate that there is a difference in LIDT values between the two types of materials of about 2× for the shortest (600-fs) and longest (1.5-ns) pulse lengths tested. The dissimilarity increases at intermediate pulse lengths (2.5 ps to 100 ps) with a maximum value greater than 3× at the 10-ps pulse length. Larger LIDT variations (20% to 50%) were also observed for unsaturated LC materials as compared to those for their saturated counterparts (5% to 11%). This larger variation in the LIDT of unsaturated materials is attributed to their increased and varying amounts of *π*-electron delocalization.

**Figure 4:**
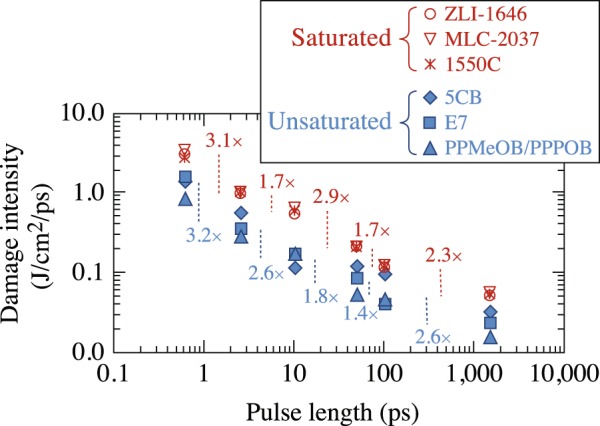
The intensity required to induce laser damage decreases with pulse length for all materials tested. The vertical lines and associated quantification factors depict the difference between the average *N*-on-1 intensities at subsequent pulse durations required to induce damage.

The LIDT results were also examined as a function of the laser intensity at each pulse length and a damage-threshold intensity was determined for each material. The results, shown in Fig. 4, quantify the difference in the damage-threshold intensity observed between successive test pulse lengths for both saturated and unsaturated materials. At the shortest pulse lengths, both types of materials undergo a similar ~3× reduction in damage threshold intensity between 600 ps and 2.5 ps. Similarly, as the pulse duration increases from 50 ps to 100 ps and from 100 ps to 1.5 ns, the average damage-threshold intensity changes by similar amounts in each increment for both saturated and unsaturated materials (~1.5× and ~2.5×, respectively). Around 10 ps, however, the damage-threshold intensity changes by differing amounts for the two material types. Specifically, between 2.5 ps and 10 ps, the change in the damage threshold intensity is lower for the *saturated* materials than for the *unsaturated* materials (1.7× and 2.6×, respectively). This difference is reversed between 10 ps and 50 ps, where the change in the damage-threshold intensity is 2.9× and 1.8× for the *saturated* and *unsaturated* materials, respectively. The possible origin of this behavior will be presented in the Discussion section.

### Wavelength dependence

The set of LC materials used in this work were also tested using nanosecond laser excitation at 351 nm (third harmonic, 3*ω*) and 527 nm (second harmonic, 2*ω*) to complement the results obtained at the fundamental 1053-nm (1*ω*) wavelength presented above. This multiwavelength investigation probed the correlation between the electronic structure of each material and its laser-induced damage behavior via altering the excitation photon energy.

**Figure 5:**
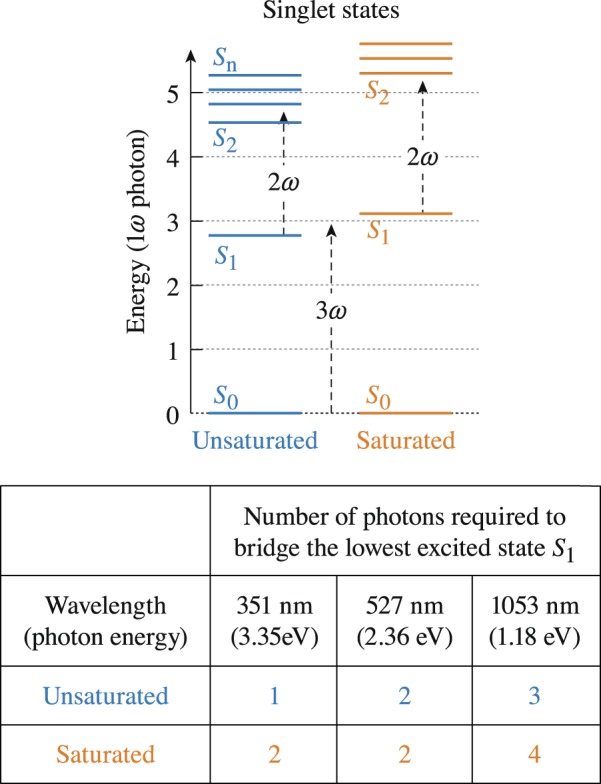
A schematic depiction of the electronic transitions leading to laser-induced breakdown in LC materials is presented by a modified Jablonski energy diagram involving a singlet ground state (*S*_0_) and excited singlet states (*S*_1_, *S*_2_,…*S*_*n*_). The energy levels are referenced as multiples of a 1053-nm photon (1*ω*). Transmission measurements for each material (see Table 1) provide insight into the order of photon absorption required to bridge the energy gap from *S*_0_ → *S*_1_.

The electronic excitation pathways in LC materials are generally known and involve a singlet ground state (*S*_0_) and excited singlet (*S*_1_, *S*_2_,…*S*_*n*_) and triplet states. The time scale of the transition from the singlet states to the corresponding triplet states during relaxation, or intersystem crossing, is typically > 1 ns, which has been confirmed for several unsaturated LC compounds^23,24^ Because the excitation leading to laser-induced damage (breakdown) occurs during the laser pulse, transitions with lifetimes longer that the pulse duration (in our case ~1 ns) have no (or minimal) effect on laser-damage mechanisms. Consequently we consider only the transitions between the singlet states. The accordingly modified Jablonski energy diagram in Fig. 5 describes the electronic structure in LC materials involving a singlet ground state (*S*_0_) and excited singlet (*S*_1_, *S*_2_,…*S*_*n*_), where the energy levels are defined as multiples of the energy of a 1*ω* photon used in this study.

The wavelengths designating the onset of linear absorption for each LC material given in Table 1 are used as a guide to suggest the order of photon absorption required for the *S*_0_ → *S*_*1*_ electronic state transition for unsaturated and saturated materials. Under 1053-nm laser irradiation, the unsaturated materials require three-photon absorption for the *S*_0_ → *S*_1_ transition, while the saturated materials require four-photon absorption. This difference in the order of the absorption process required to generate excited-state electrons is captured clearly by the difference in the damage threshold between the two types of materials, where the saturated materials have 2× to 3× higher damage threshold across all pulse lengths tested (Figs 1 and 3).

### Laser-induced damage testing at 351 nm

**Figure 6:**
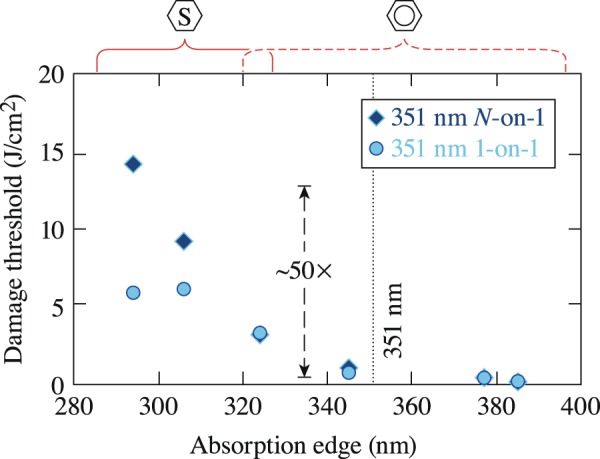
LIDT results for saturated and unsaturated LC materials irradiated with 351-nm and 1.0-ns pulses. The large difference in the LIDT between the saturated and unsaturated materials is attributed to a change in the order for the *S*_0_ → *S*_1_ transition. The 1-on-1 marker lays almost exactly on top of the N-on-1 marker for several materials.

The LIDT results shown in Fig. 6 indicate that the laser-induced–damage thresholds under irradiation with 351-nm and 1-ns pulses follow the absorption edge of the LC materials, a trend that is particularly clear for the *N*-on-1 results. This behavior may be assigned in part to the order of the excitation process. As depicted in the schematic representation of the energy structure of the saturated and unsaturated materials shown in Fig. 6, the highly unsaturated materials require one-photon absorption for the *S*_0_ → *S*_1_ transition, while saturated materials require a two-photon absorption process. This difference is potentially responsible for the large difference in the LIDT (~20× to 50×, depending on damage testing method) between the saturated and unsaturated materials. The results also show that laser conditioning (*N*-on-1 > 1-on-1 LIDT) occurs in saturated materials. This issue will be discussed in more detail in the Discussion section.

### Laser-induced damage testing at 527 nm

**Figure 7:**
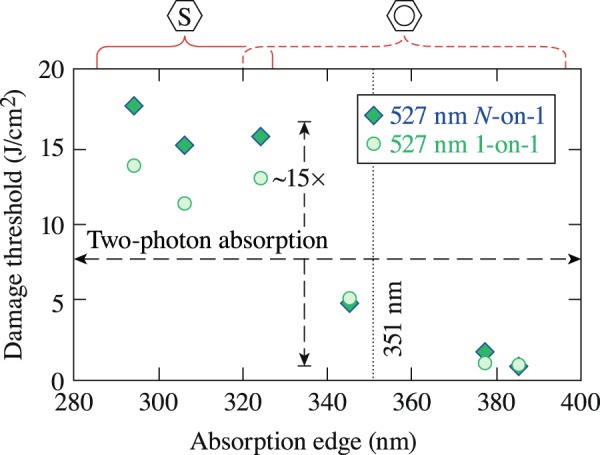
LIDT results for saturated and unsaturated LC materials irradiated with 527-nm and 1.2-ns pulses. Because all of the LC materials evaluated require two-photon absorption for the *S*_0_ → *S*_1_ transition, it is hypothesized that the strong dependence on material saturation arises from the different orders of excited-state absorption required to reach the next electronic-state transition.

The LIDT results under irradiation with 527-nm and 1.2-ns pulses are shown in Fig. 7. Based on the absorption characteristics of all materials, both unsaturated and saturated materials require two-photon absorption to populate the first excited state. Therefore, the strong dependence of the LIDT on material saturation (~10× to 15× difference) cannot be assigned to the *S*_0_ → *S*_1_ transition cross sections. We hypothesize that this behavior arises from differences in the absorption cross section for transition between excited states *S*_1_ → *S*_2_. Specifically, we hypothesize that the saturated materials require two-photon absorption for the *S*_1_ → *S*_2_ transition, while unsaturated materials need only one-photon absorption. This difference in the electronic excitation process is depicted in the schematic representation of the electronic energy level diagram shown in Fig. 5. Previous studies of the excited-state absorption spectrum in different LC materials revealed that the energy separation between the first two excited-states can be either higher or lower than the 527-nm photon energy, depending on the material^23,24^. Significant laser conditioning is once again observed in the saturated materials.

### Damage testing at 1053 nm

The LIDT results under irradiation with 1053-nm, 1.5-ns pulses were shown previously in Fig. 1(a). The difference in the LIDT between saturated and unsaturated materials can be assigned to the change in the order of the excitation process for the *S*_0_ → *S*_1_ transition (three-photon versus four-photon absorption, respectively). This difference in the order of the absorption process required to generate excited-state electrons is clearly captured by the difference in the LIDT between the two types of material. Although the difference in the 1053-nm LIDT between saturated and unsaturated materials is 2× to 3× higher across all pulse lengths tested, these values are relatively small compared to the difference in LIDT between the two material systems observed with 527-nm (~15×) and 351-nm pulses (~50×).

## Discussion

The results demonstrate that the LIDT values exhibit a strong dependence on the incident laser wavelength, which indicates that damage initiation is sensitive to the energy separation between the ground and excited states. To deposit a sufficient amount of energy to initiate damage, electrons must be excited to a level *S*_*n*_ ≥ *S*_2_. Among all probable pathways for the *S*_0_ → *S*_*n*_ transition, the one that involves the lowest-order excitation processes (through existing intermediate states) is expected to be the dominant mechanism. In our system, this principle implies that the electrons will first undergo the *S*_0_ → *S*_1_ transition, followed by the *S*_1_ → *S*_2_ transition, and then followed by possible single-photon transitions to reach higher excited states (due to smaller energy separations).

Upon excitation of electrons to the first excited state, additional excited-state absorption will require a lower-order absorption process because the energy separation between *S*_1_ and *S*_*n*_ states is smaller compared to that between *S*_0_ and *S*_1_ states^23,24^. This smaller energy separation, in turn, yields a higher absorption cross section for excited-state absorption (ESA) and is therefore critical in the context of laser damage. Because the lifetime of the *S*_1_ state is of the order of 1 ns or longer^23,24^ in this work, the excited electrons do not return to the ground state during the laser pulse. Consequently, ESA is a more effective energy-deposition mechanism, but it is limited by the available excited-state electron population. We therefore consider two general governing mechanisms that contribute to absorption of energy by the laser pulse: (a) direct absorption by ground-state electrons and (b) absorption by excited-state electrons involving only the singlet states. The excited-state electrons can, in principle, undergo multiple absorption cycles by either reaching the higher excited state (*S*_2_) and returning to the *S*_1_ state during the laser pulse to repeat the process or continuing with additional absorption toward higher excited states (*S*_2_ → *S*_*m*_).

Laser-induced damage experiments that capture pulse-length scaling can contain information on energy-deposition mechanisms. Of particular interest is the change in the normalized LIDT between the two types of materials at 10 ps (Fig. 3), which transitions from a twofold to a threefold increase before returning to initial values at longer pulse lengths. This change is also captured in Fig. 4 in the same pulse-length regime by the very different quantification factors between LIDT intensity at successive pulse lengths. The behavior observed in Figs 3 and 4 in the range from 3 to 50 ps likely arises from the complex interplay between energy-deposition mechanisms, as well as the temporal behavior of the excited-state absorption rate. Specifically, O’Keefe^24^ showed that the absorption rate for CB15 (an unsaturated cyanobiphenyl identical in structure with 5CB except that it has a *chiral* alkyl terminal group instead of the straight-chain alkyl group found on 5CB) can change rapidly within time scales of the order of 10 to 50 ps, which could directly impact the average efficiency (rate of energy deposition) of ESA as a function of the laser pulse length. An additional consideration that could contribute to the behavior at 10 ps in the *N*-on-1 regime would be the impact of laser conditioning, which our work has demonstrated to be pulse-length dependent (discussed later and shown in Fig. 9).

**Figure 8:**
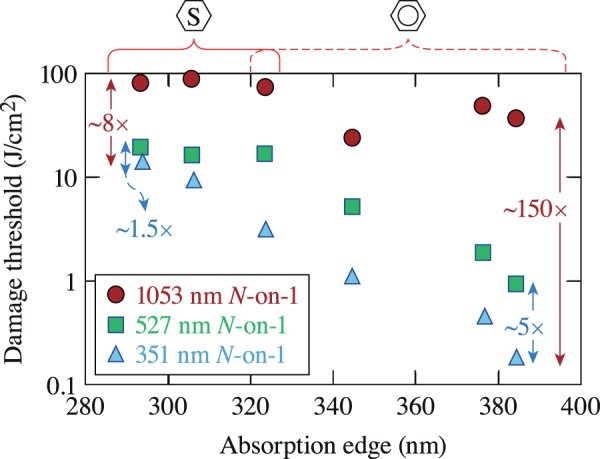
The *N*-on-1 LIDT values for nanosecond pulses at all three wavelengths as a function of each material’s absorption edge allows direct comparison of the relative differences in LIDT.

To better illustrate the relative difference in the measured LIDT at different wavelengths, the *N*-on-1 LIDT results for both saturated and unsaturated materials at all wavelengths are shown in Fig. 8. Comparison of the 351-nm and 527-nm results shows a difference between LIDT values of ~5× for unsaturated materials and only of ~1.5× for saturated materials. This behavior can be anticipated from the order of absorption required for electrons to undergo the *S*_0_ → *S*_1_ transition. Specifically, unsaturated materials require both linear absorption at 351 nm *and* two-photon absorption at 527 nm, while for saturated materials, two-photon absorption is necessary to populate the first excited state at *both* wavelengths. This key difference in the electronic excitation process is reflected in the corresponding relative difference in the LIDT values.

Comparing LIDT results obtained under 351-nm and 1053-nm excitation, the difference in LIDT for unsaturated materials is ~150× but only ~8× for saturated materials. The dramatic variation in LIDT differences for the two material types is arguably related to the different order of the absorption process required for the *S*_0_ → *S*_1_ transition. The order changes from linear absorption to a three-photon absorption process in unsaturated materials, while for saturated materials a nonlinear process is required at both wavelengths (two-photon and four-photon processes for 351-nm and 1053-nm excitation, respectively). These results demonstrate the importance of the electronic structure of each material on the observed damage threshold and as a function of photon energy.

**Figure 9:**
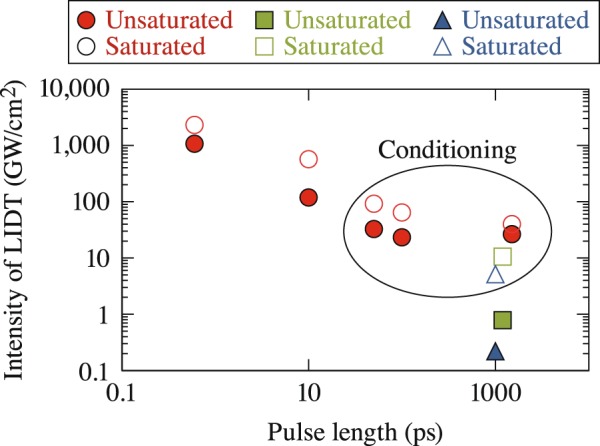
The average damage intensity, based on 1-on-1 LIDT values, for saturated and unsaturated materials is shown for each tested pulse length and wavelength. The results suggest the laser conditioning appears at laser intensities higher than ~5 GW/cm^2^. The data also show a lower bound for the time scale (<50 ps) below which laser conditioning is not observed.

Laser conditioning (*N*-on-1 LIDT > 1-on-1 LIDT) was observed only under a subset of conditions: (a) both material types at 50 ps, 100 ps, and 1.5 ns at 1053 nm and (b) saturated materials at 527 nm and 351 nm. The mean 1-on-1 LIDT values for both material types were determined by averaging experimental results for unsaturated LC’s (5CB, E7, and the PPMeOB/PPPOB mixture) and saturated LC’s (1550C, MLC-2037, and the partially saturated ZLI-1646) at each wavelength. The corresponding intensity for each material type was plotted as a function of the pulse length in Fig. 9. The subset of data associated with laser conditioning is noted.

The highlighted data associated with observations of laser conditioning shown in Fig. 9 are found in the upper right quadrant with intensities higher than ~5 GW/cm^2^ and pulse duration longer than 10 ps. This observation suggests that a threshold intensity exists for laser conditioning. Unsaturated compounds have LIDT values lower that this conditioning threshold intensity (<5 GW/cm^2^) at both 351 nm and 527 nm. Results also suggest that laser conditioning is limited to time scales greater than 10 ps. There are two scenarios for this limitation: In one case, conditioning involves a two-step process with a characteristic rise time (of the order of 10 ps) associated with the promotion of electrons to an excited state, followed by transition to an intermediate state where further excitation can facilitate conditioning such as via migration of the electron into a different site where it exhibits lower absorption. In the second case, the laser conditioning mechanism is dictated by the time scale of photochemically induced reaction kinetics [e.g., volatilization of impurities or photochemically induced reaction of the LC molecules with intrinsic or extrinsic impurities and/or breakdown products, or with each other to form more stable compounds (e.g., oxygen bridge formation in biphenyls)]. Further studies are required to understand the origin and mechanism of laser conditioning in LC materials.

## Methods

### Sample preparation

Samples constructed for short-pulse damage testing utilized a 100-*μ*m demountable fused-silica spectroscopy cell (Starna Cells) for the bottom substrate and 150-*μ*m-thick fused-silica microscope cover slips (Technical Glass Products) for the top substrate to minimize self-focusing effects. For testing at nanosecond pulse lengths, sandwich cells filled with LC were constructed using two 10-mm-thick fused-silica substrates and 100-*μ*m glass bead spacers. The depth of the demountable cells was confirmed to be 100 *μ*m ± 5 *μ*m, and the uniformity of the sandwich cell gaps were of the same order. Antireflection coatings were not used in these devices. The LIDT of the substrates was first measured separately to ensure that the measured values of the tested samples are not a result of damage on the substrates. All devices were constructed and sealed using UV-curing epoxy (Master Bond #UV15-7TK1A). The advantage of using a relatively large 100-*μ*m cell gap in these experiments, rather than thinner cells with good LC molecular alignment (of fundamental importance for device development), is that it enables one to determine the intrinsic bulk LC LIDT resulting from interaction of the laser energy with the LC molecules exclusively and eliminates boundary condition effects such as damage initiation by chemical/photochemical interaction of the LC with the alignment layer, epoxy sealing material, or defects on the substrate surfaces. The results reported here should be considered to represent each LC material’s bulk behavior for the net average molecular orientation independent of cell boundary conditions.

All test cells were assembled using LC materials that had been first purged with high-purity helium followed by filtration through a 0.2-*μ*m Teflon membrane filter. The devices were heated to 100 °C on a hot plate and filled by capillary action while the LC was in the isotropic state. Two sets of samples for each material were prepared to ensure that fresh, uncontaminated materials were tested over the time span of the measurements.

### Laser system

The nanosecond damage testing system is based on a Nd:YLF *Q*-switched laser with an injection seeding laser system operating at 1053 nm. The laser system is also equipped with harmonic generation crystals to produce pulses at 527 nm and 351 nm. Experiments were performed using pulses with nearly Gaussian temporal profiles and duration at a full-width-at-half-maximum pulse duration of 1.5 ns, which yields pulses of about 1.2 ns and 1 ns when operating at the second- and third-harmonic wavelengths, respectively. This system has an average 1/*e*^2^-beam spot size of 600 *μ*m at a 7° incidence angle. The short-pulse damage testing system (0.6 to 100 ps) at 1053 nm has an average 1/*e*^2^-beam spot size of 350 *μ*m incident at 45°. At this angle, if damage due to self-focusing effects has occurred at the exit substrate, it can be easily differentiated from bulk sample damage. All samples were tested in air using *s*-polarized light. Since damage testing was performed using unaligned LC samples, the angle of incidence did not affect the test results in the same manner as it would have for aligned samples.

### Laser-induced damage testing protocol

Both 1-on-1 (single shot per test site) and *N*-on-1 (up to ten shots at 0.1 Hz, with the fluence ramped until damage occurs) damage testing were performed to determine the corresponding LIDT. Fresnel losses and the angle of incidence were both accounted for in the LIDT values. Because the LC mesophase is fluid, a test site is considered damaged upon any visual change, as observed with an *in-situ* long-working-distance imaging system (Infinity Photo-Optical Company K2/SC long-distance microscope system) with a 2.5-*μ*m resolution. Due to the unaligned nature of the LC samples, materials with a larger birefringence (unsaturated compounds) exhibit higher scattering of the illumination light, which increases the difficulty of directly detecting a damage event. To address this issue, the sample illumination and image acquisition were adjusted to avoid pixel saturation in the direct image, while damage was determined from a differential image generated by subtracting a pre-exposure image (taken before testing was initiated) from post-exposure images acquired immediately after laser exposure. Damage-induced changes (typically bubbles, which often look like spots) were detected by the change of scattered light over a micron-scale area. This damage behavior is analogous to the bubble formation observed in the liquid environments of medical applications^25^, where the expansion of gaseous products produced during the laser interaction creates a bubble in the liquid. The bubbles typically redissolve or migrate on a time scale dependent on laser fluence, fluid viscosity, and solubility of the gas in the fluid medium.

The number density and size of these features increased with increasing fluence above the damage threshold. Although this behavior suggests a “healing” effect, products of the LC/laser interaction, such as gases (which form bubbles) or carbonaceous residues, remain within the sample volume (at least for a limited amount of time in the case of bubbles) and could affect subsequent results. To address this issue, samples were mounted vertically and new test sites were selected below previously tested sites to prevent migrating bubbles from impacting the measurement results. Additional details on sample preparation, testing protocol, and examples of damage morphology are found in ref.^26^.

## Conclusion

In summary, this report investigates the laser breakdown threshold for a series of saturated and unsaturated nematic LC materials at pulse lengths between 600 fs and 1.5 ns at 1053 nm as well as in the nanosecond pulse range at 527 nm and 351 nm. The observed LIDT values show a strong dependence on wavelength and electronic structure, which in turn provides information about the excitation pathways leading to laser-induced damage. As a result, saturated materials invariably exhibit higher LIDT’s. Experimental data suggest that key components in the laser-induced damage mechanisms in LC’s involve a complex interplay of both multiphoton absorption and excited-state absorption, where their relative contributions vary with both pulse length and wavelength. Future work will concentrate on extending and applying these findings to both glassy and polymer LC materials systems and developing improved passive and active devices that offer polarization, phase, and intensity control for high-peak-power and average-power laser applications.
